# First-in-Human Experience With Septal Occluder for Dehiscence of Anterior Mitral Valve Leaflet Patch: From Surgical to Percutaneous Closure

**DOI:** 10.1016/j.jaccas.2025.105291

**Published:** 2025-10-22

**Authors:** Alexandru Patrascu, Rami Abazid, Mohammed Alkasab, Thomas Attumalil, Amr Gamal, Bryan Traynor, Bobby Yanagawa, Kendra Derry, Sami Alnasser, Neil P. Fam

**Affiliations:** a Structural Heart Program, St Michael's Hospital, University of Toronto, Toronto, Ontario, Canada; b Cardiology Division, Sault Area Hospital, Northern Ontario School of Medicine University, Sault Ste Marie, Ontario, Canada; c Division of Cardiac Surgery, St Michael's Hospital, University of Toronto, Toronto, Ontario, Canada

**Keywords:** device closure, endocarditis, patch dehiscence, perforation, mitral regurgitation

## Abstract

**Background:**

Mitral regurgitation secondary to infective endocarditis is often due to structural damage to the leaflets or apparatus, and the condition warrants surgical treatment. If surgery fails, percutaneous repair may be a viable option.

**Case Summary:**

We describe a case of anterior mitral valve leaflet perforation after infective endocarditis, initially treated by surgical patch repair, with subsequent dehiscence leading to recurrence of severe mitral regurgitation. After percutaneous closure with a septal occluder device, regurgitation was abolished.

**Discussion:**

To our knowledge, this is the first case to demonstrate use of the Gore Cardioform septal occluder to treat postsurgical dehiscence of anterior mitral valve leaflet patch. Device selection needs to be decided on a case-by-case basis.

**Take-Home Message:**

Percutaneous treatment of post–infective endocarditis anterior mitral valve leaflet perforation is feasible, even after failed surgical repair.

## Graphical Abstract

**Figure:**
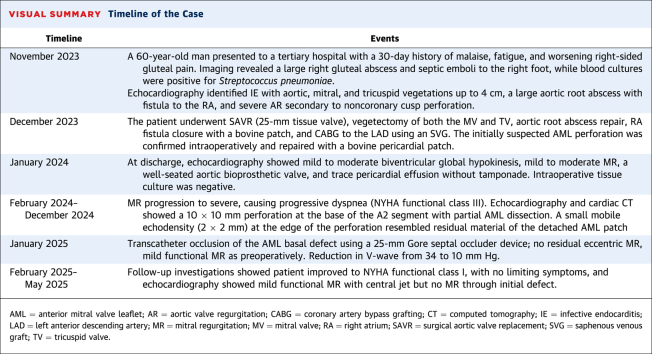

## History of Presentation

A 61-year-old man was referred for potential transcatheter repair of chronic anterior mitral valve leaflet (AML) perforation after infective endocarditis (IE) 1 year prior. Progression of mitral regurgitation (MR) over time led to NYHA functional class III dyspnea and persistent fatigue, which warranted evaluation by our structural heart team. Physical examination at presentation showed a typical pansystolic murmur at the apex, and no evidence of edema. The patient had no recent heart failure hospitalizations, but he was symptomatic despite medical therapy with 2.5 mg bisoprolol, 5 mg dapagliflozin, 25 mg spironolactone, 20 mg furosemide, and 40 mg valsartan daily.

**Take-Home Messages**

• Percutaneous treatment of post–infective endocarditis anterior mitral valve leaflet perforation can be considered an option, even after failed surgical patch repair.
• Device choice needs to be assessed on an individual basis based on defect location and risks of interference with aortic valve function and hemolysis.

## Past Medical History

Twelve months earlier, the patient had presented to a tertiary hospital with a 30-day history of malaise, fatigue, and worsening right-sided gluteal pain. He was febrile with elevated inflammation markers. Imaging had revealed a large right gluteal abscess, treated by drain insertion. However, he developed septic emboli to the right foot and positive blood cultures for pansensitive *Streptococcus pneumoniae*. Except for remote left-sided hip replacement, there was no further medical history, and no IE-predisposing condition.

**Figure 1:**
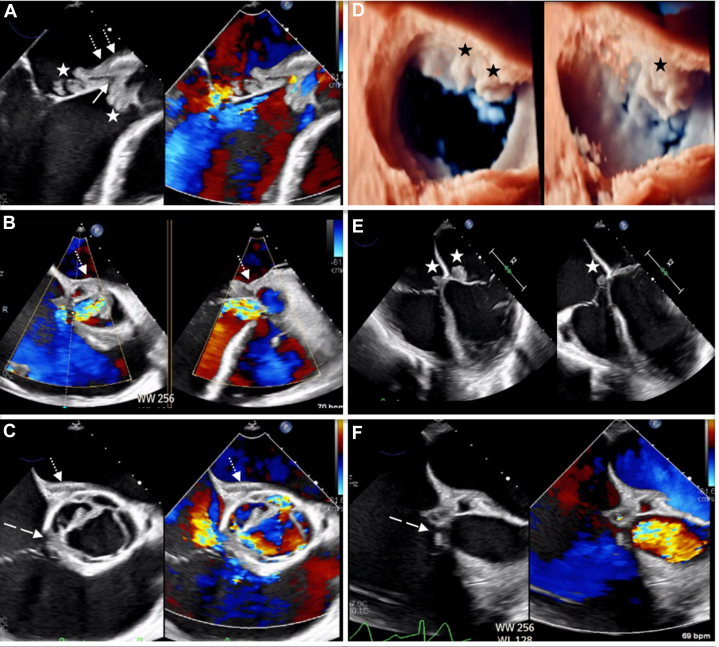
Preoperative Transesophageal Echocardiography (A, B, C, and F) Images show a large aortic root abscess (dotted arrows) causing noncoronary cusp perforation, right atrial fistula (dashed arrows), and severe aortic regurgitation. Also, multiple vegetations (stars) of the noncoronary aortic leaflet, anterior mitral valve leaflet, and septal tricuspid valve leaflet (A, D, and E) are seen, while perforation of the base of the anterior mitral leaflet is suspected (solid arrow, panel A).

Transthoracic echocardiography (TTE) and transesophageal echocardiography (TEE) identified multivalvular endocarditis with aortic, mitral, and tricuspid vegetations up to 4 cm, a large aortic root abscess with fistula to the right atrium, and severe aortic regurgitation secondary to noncoronary cusp perforation (Figure 1, Video 1). Angiography showed 80% mid left anterior descending artery stenosis, although left ventricular ejection fraction (LVEF) was preserved. The patient eventually underwent complex cardiac surgery including aortic valve replacement (25-mm tissue valve), vegetectomy of both mitral and tricuspid valves, aortic root abscess repair, right atrial fistula closure with a bovine patch, and coronary artery bypass grafting to the left anterior descending artery using a saphenous vein graft. Also, after the large abscess cavity underneath the aortic valve was revealed, between the aortomitral curtain and the fistulous process into the right atrium, the initially suspected AML perforation (Figure 1) became evident and was repaired with a bovine pericardial patch.

Postoperatively, the patient required a dual-chamber pacemaker for complete heart block. His postsurgical TTE showed mild to moderate biventricular global hypokinesis, with mild left ventricular (LV) dilatation (left ventricular end-diastolic volume index [LVEDVi]: 104 mL/m^2^, left ventricular end-systolic volume [LVESD]: 45 mm), mild to moderate functional MR and tricuspid regurgitation, a well-seated aortic bioprosthetic valve, and trace pericardial effusion without tamponade. Intraoperative tissue culture was negative. The patient remained afebrile on ceftriaxone, his infection parameters normalized, and several magnetic resonance imaging investigations and orthopedics consultations cleared concerns of other infection sources. He was discharged and completed a 6-week course of intravenous ceftriaxone in the community.

## Differential Diagnosis

Follow-up investigations showed improvement in LVEF from 40% to 50% at 1 year. However, progression of both MR and LV size (LVEDVi: 111 mL/m^2^, LVESD: 47 mm), together with worsening dyspnea, were noted. Moreover, TTE demonstrated a recurrent MR jet at the base of the AML, raising concerns of possible patch dehiscence or recurrent IE.

## Investigations

**Figure 2:**
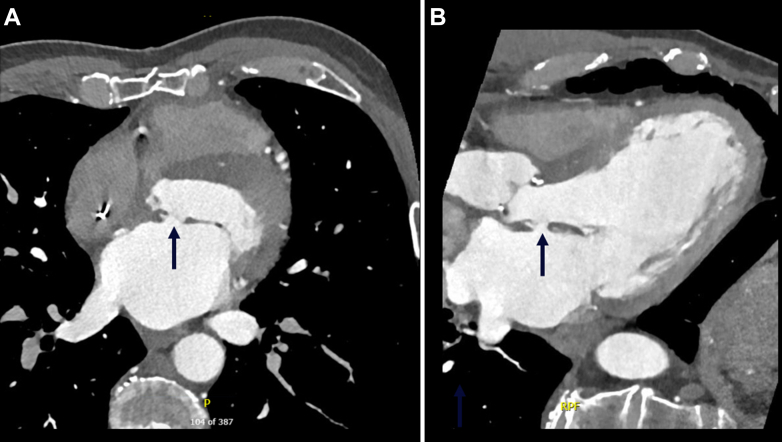
Preprocedural Cardiac Computed Tomography (A) Axial plane and (B) sagittal plane computed tomography reveal a 10 × 10 mm defect (arrows) of the basal anterior mitral valve leaflet, despite patch repair 1 year prior.

**Figure 3:**
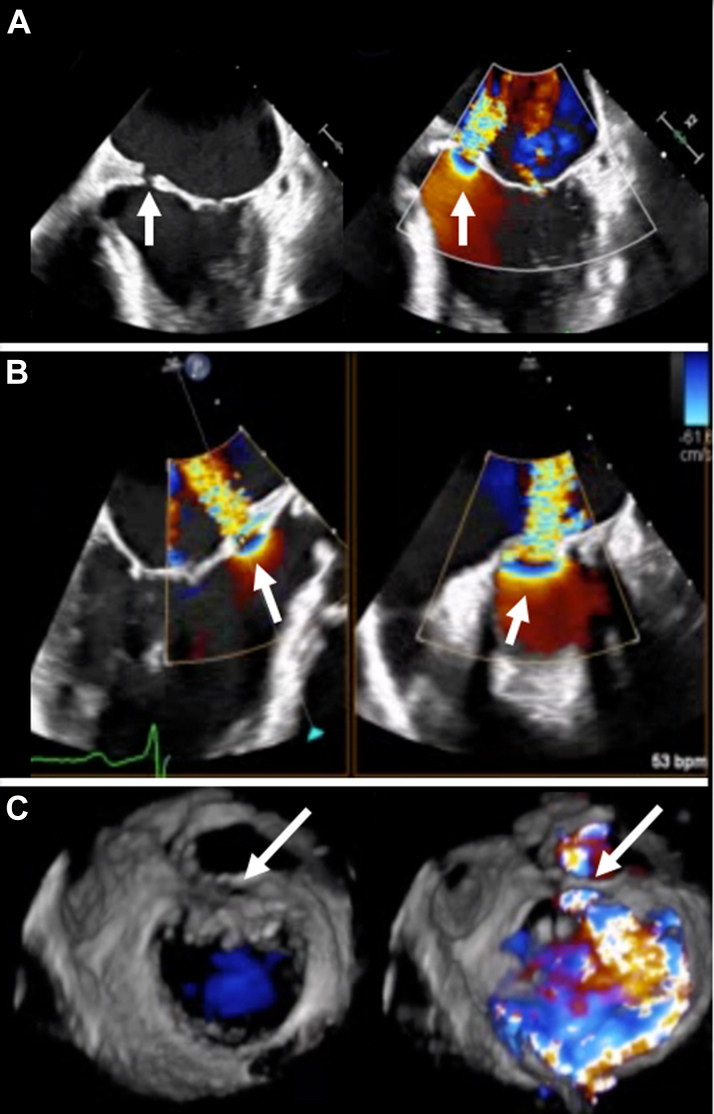
Preprocedural Transesophageal Echocardiography (A and B) Biplane and (C) three-dimensional imaging confirm a basal A2 segment defect (arrows) after dehiscence of a previous bovine pericardial patch, causing severe mitral regurgitation.

Electrocardiography revealed normal sinus rhythm at 60 beats/min, and pacemaker interrogation showed <1% ventricular pacing. Three sets of blood cultures were negative, with no evidence of hemolysis. Left heart catheterization showed a patent saphenous vein graft to the left anterior descending artery, and LV angiography confirmed mild systolic function impairment in the presence of severe MR. Next, on cardiac computed tomography, the bioprosthetic aortic valve appeared well-seated with normal thickness of leaflets, but there was a 10 × 10 mm perforation of the basal AML (Figure 2). This was confirmed on TEE to be the A2 segment, causing severe MR (proximal isovelocity surface area: 12 mm, effective regurgitant orifice area: 160 mm^2^) through the defect (Figure 3, Video 2). Blood/contrast flow within the body of the leaflet on each respective imaging method was suggestive of partial AML dissection. Moreover, a small mobile echodensity (2 × 2 mm) at the edge of the perforation suggested residual material of the dehisced AML patch (Video 2).

## Management

**Figure 4:**
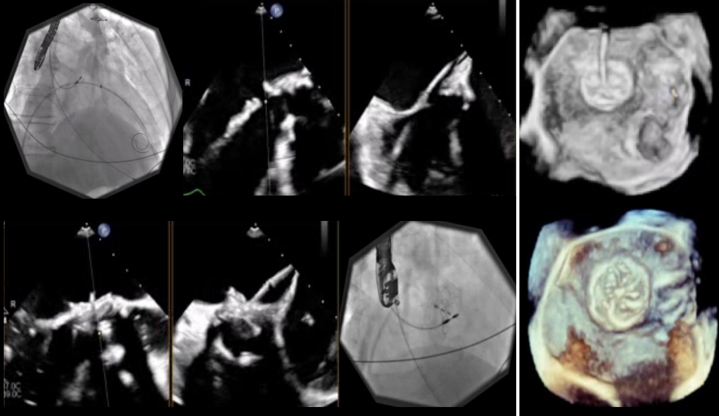
Procedural Steps for Antegrade Deployment of the Gore Cardioform Device Steps include (Upper Left Panel) crossing the anterior leaflet defect to (Lower Right Panel) deployment of both discs. (Far Right Panels) Stable position and no residual flow are seen on three-dimensional imaging.

The case was discussed at our heart team rounds. As the patient was turned down for repeat surgery, the proposed plan was percutaneous device closure to address the AML defect. Under general anesthesia, the right common femoral vein was cannulated, an 18-F Gore sheath (W.L. Gore) was placed, trans-septal access to the left atrium was established with the VersaCross system (Boston Scientific), and septostomy with a Mustang 6-mm balloon (Boston Scientific) allowed advancement of an Agilis small-curl catheter (Abbott Vascular) into the left atrium, which was then steered toward the AML defect. Next, a Glidewire (Terumo) crossed the AML defect into the LV, and was then exchanged for an Amplatz Extra Stiff wire (Cook Medical) over a 4-F multipurpose catheter (Cordis). Initially, a 20-mm Gore Cardioform septal occluder^1^ (W.L. Gore) was advanced through the defect and deployed. As this device did not provide complete seal, it was exchanged for a 25-mm Gore Cardioform septal occluder, which eliminated the MR and showed stable position after a tug test (Figure 4, Videos 3 and 4). The mean gradient was 4 mm Hg, and there was no change in aortic valve function. In addition, the left atrial V-wave was reduced from 34 to 10 mm Hg. The patient was extubated and was transferred to the recovery room in stable condition.

## Outcome and Follow-Up

**Figure 5:**
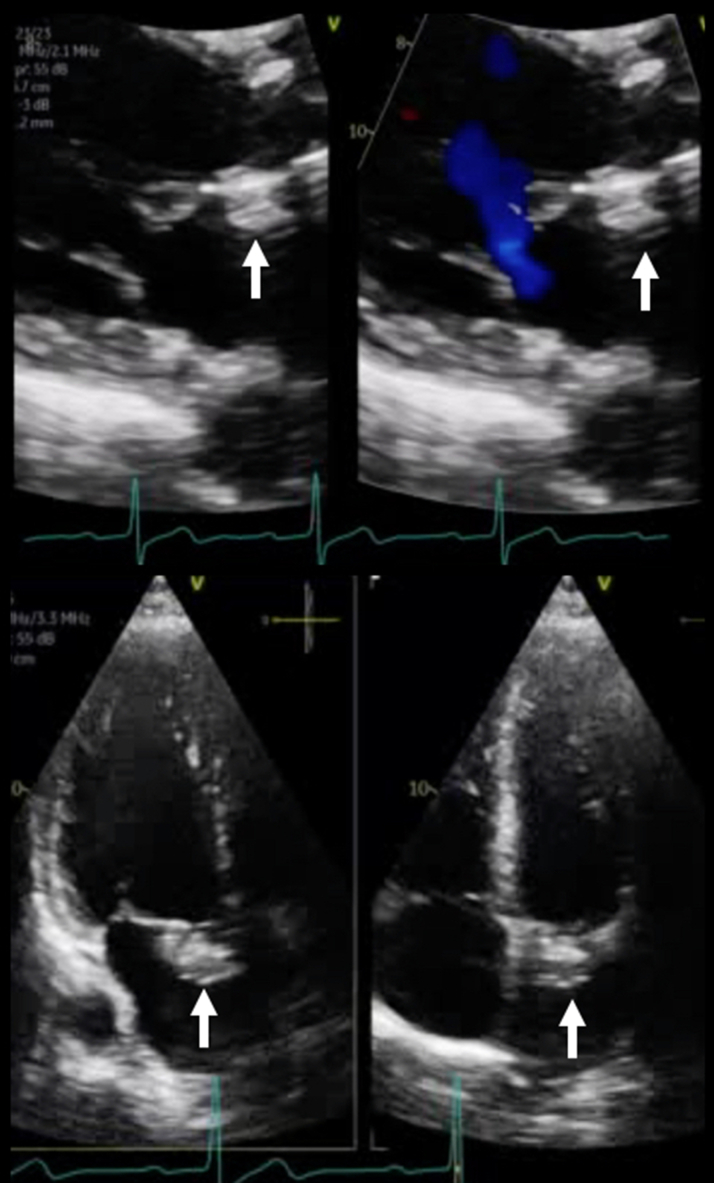
Follow-Up Transthoracic Echocardiography Five-month follow-up images confirm stable device position (arrows), with no residual leak and mild functional mitral regurgitation.

The patient's hospital course was uneventful, with no complications. He was started on clopidogrel 75 mg daily for 6 months, in addition to lifelong aspirin and existing heart failure therapy. Both discharge and 5-month follow-up TTE investigations confirmed stable position of the Gore device, with mild residual functional MR, mean gradient of 4 mm Hg, unchanged mildly reduced LV systolic function with reverse remodeling (LVEDVi: 102 mL/m^2^, LVESD: 44 mm), and no interference with the bioprosthetic aortic valve or LV outflow tract (Figure 5, Video 5). Furthermore, the patient improved to NYHA functional class I symptoms at follow-up and confirmed significant improvement in quality of life.

## Discussion

AML perforations are very rare, with a scarcity of reports published in the literature. Although there are various etiologies of AML defects, including iatrogenic complication of aortic valve replacement^2^ or TAVR,^3^ repair of atrioventricular septal defects, and IE,^4^ the primary treatment is usually surgical repair. Sareyyupoglu et al^5^ reported 26 patients with AML perforation who underwent mitral valve repair, of whom 24 patients (92%) had endocarditis. For AML repair, a patch was used in 11 patients (42%) and primary suture closure in 15 patients (58%). The 1-year survival rate was 95%. As encouraging as these surgical outcomes are, one needs to keep in mind that in the presence of IE, repair is being performed on friable mitral valve tissue, as was the case in our patient. This may affect long-term results, for example, leading to dehiscence of an AML patch, which raises the question of repeat surgery.

For patients turned down for surgical AML repair, whether as first or redo procedure, transcatheter defect closure seems to be an acceptable alternative in the absence of active infection. So far, very few reports are available, most using the Amplatzer septal occluder (St Jude Medical). However, mixed results are available, from complete sealing,^6^ to residual MR through the device,^7^ to severe hemolysis.^8^ To our knowledge, an alternative device such as the Gore septal occluder was only used in 4 patients. In a previously published mini-series of 3 cases,^9^ sealing of AML perforation in nonsurgical, non-IE patients with the Gore occluder, in conjunction with the MitraClip system (Abbott) to stabilize the mitral valve leaflets, led to 1 successful procedure, 1 death, and 1 deformation of discs necessitating retrieval. Another report^8^ on its use as bailout after Amplatzer-related hemolysis, in an IE case initially turned down for surgery, led to unlocking of the left atrial disc after deployment and emergent surgical repair. The discrepancies in outcomes across published cases can be attributed to multiple factors, including device properties (profile, material, size), anatomical challenges (defect location and size, access route, interference with the aortic valve), team expertise (both interventional and imaging guidance), and patient-specific variables (individual risk factors for infection or hemolysis).

Our case differed in that the patient had a late surgical complication of patch dehiscence, and there was also evidence of leaflet dissection. Although degradation by resorption or dissolution was theoretically possible, this seemed extremely unlikely, as the same bovine pericardial patch was used to close the aortic root and the right atrial fistula. Therefore, we felt that the Gore Cardioform was best suited for our patient owing to its unique construction. Whereas cribriform devices are made of nitinol mesh, with larger gaps allowing rapid acceleration of the jet, it seems possible that the Cardioform device is associated with better seal and less hemolysis^10^ in these cases of high-pressure jets, owing to an occlusive expanded polytetrafluoroethylene membrane.

Other technical considerations that need to be addressed are whether to advance the occluder through an antegrade trans-septal^6^ or retrograde transaortic approach,^7^ while being mindful of possible aortic valve obstruction. As our patient already had a bioprosthetic aortic valve in place with slightly elevated gradients (mean: 20 mm Hg), and the defect was at the base of the A2 segment, we thought an antegrade approach would allow for proper defect engagement while avoiding injury to the aortic bioprosthesis. Furthermore, preprocedural planning by TEE and computed tomography also helped narrow the device size and choice (20-mm/25-mm Gore Cardioform septal occluder vs 12-mm/14-mm Amplatzer septal occluder) while paying respect to the distance to both the aortic valve bioprosthesis and the mitral valve coaptation line, in order to avoid iatrogenic valvular stenosis. Lastly, cerebral protection devices could be considered in cases with large calcified defects, mechanical valves, or in patients with prior strokes.

## Conclusions

AML perforation secondary to IE warrants prompt surgical treatment. Here, we demonstrate the feasibility of percutaneous closure of late AML patch dehiscence with the Gore Cardioform septal occluder. Whether to use an Amplatzer nitinol cribriform device or one with an expanded polytetrafluoroethylene membrane such as the Cardioform needs to be decided on a case-by-case basis.

## Funding Support and Author Disclosures

Dr Fam has been a consultant to Edwards Lifesciences, Abbott, Cardiovalve, Medtronic, Tricares, inQB8, and Jenscare. All other authors have reported that they have no relationships relevant to the contents of this paper to disclose.

## Appendix

Video 1 Preoperative Transesophageal Echocardiography Multivalvular endocarditis with aortic, mitral, and tricuspid vegetations up to 4 cm, severe aortic regurgitation secondary to noncoronary cusp perforation, and a large aortic root abscess with fistula to the right atrium. In retrospect, a basal anterior mitral valve leaflet defect can be suspected, though mostly covered by the big vegetation.

Video 2 Preprocedural Transesophageal Echocardiography Confirms a Basal A2 Segment Defect After Probable Dehiscence of a Previous Bovine Pericardial Patch, Causing Severe Mitral Regurgitation.

Video 3 Procedural Transesophageal Echocardiography to Guide the Crossing of the Anterior Mitral Valve Leaflet Defect, the Deployment of the Device, and Confirm Its Position.

Video 4 Fluoroscopic Guidance of Anterior Leaflet Defect Closure.

Video 5 Five-Month Follow-Up Confirms Stable Device Position With No Residual Leak and Mild Functional Mitral Regurgitation.

Video Summary Anterior mitral valve leaflet perforation - from surgical to percutaneous treatment
